# Supplementary material for: Poly(ADP) ribose polymerase promotes DNA polymerase theta-mediated end joining by activation of end resection
Source: Nat Commun. 2022 Aug 4;13:4547. doi: 10.1038/s41467-022-32166-7 (PMC9352658; doi:10.1038/s41467-022-32166-7)
Supplement: Supplementary file 1 — Supplementary Information [file 41467_2022_32166_MOESM1_ESM.pdf]

Supplementary Information for:

Poly(ADP) ribose polymerase promotes DNA polymerase theta-mediated end joining by activation of end resection

Megan Luedeman, Susanna Stroik, Wanjuan Feng, Adam J Luthman, Gaorav P Gupta, and Dale A Ramsden

Supplementary Figures

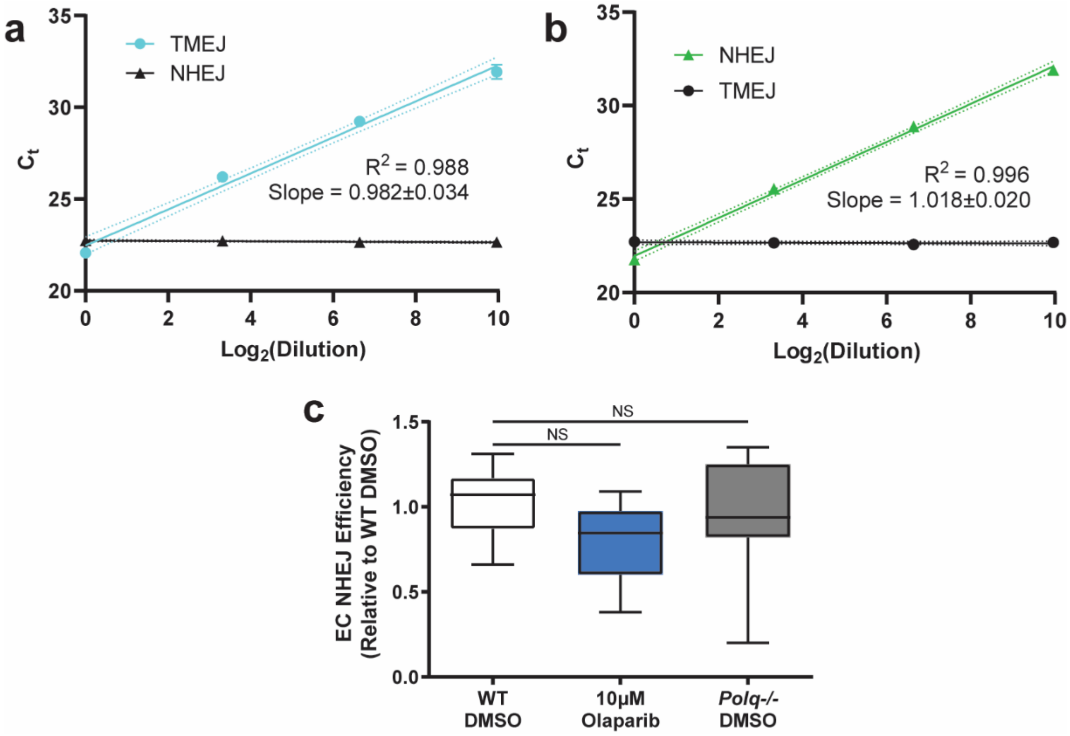

Supplementary Figure 1 Validation of linearity, limit of detection, and independence of multiplexed qPCR for extrachromosomal repair

a) Cycle thresholds (C_t) after qPCR of the TMEJ extrachromosomal (EC) amplicon (blue) serially diluted in DNA containing constant amounts of the NHEJ EC amplicon (black); data shown are mean ± SD (n=3). The slope, coefficient of determination (R^2), fitted line (solid blue line), and 95% confidence intervals (dashed blue lines) after simple linear regression of TMEJ data are noted. **b)** Cycle thresholds (C_t) after qPCR of the NHEJ EC amplicon (green) serially diluted in DNA containing constant amounts of the TMEJ EC amplicon (black); data shown are mean ± SD (n=3). The slope, coefficient of determination (R^2), fitted line (solid green line), and 95% confidence intervals (dashed green lines) after simple linear regression of NHEJ data are noted. **c)** Box (interquartile range) and whisker (upper and lower extremes) plot of the non-normalized EC joining efficiency of the NHEJ substrate in vehicle or olaparib treated WT MEFs or *Polq-/-* MEFs as a fraction of the efficiency in vehicle-treated WT cells for all EC trials (WT DMSO n=15; WT 10µM Olaparib n=12; *Polq-/-* n=15). Statistical significance of differences, relative to WT DMSO, was determined by one-way ANOVA (NS, p>0.43, not significant).

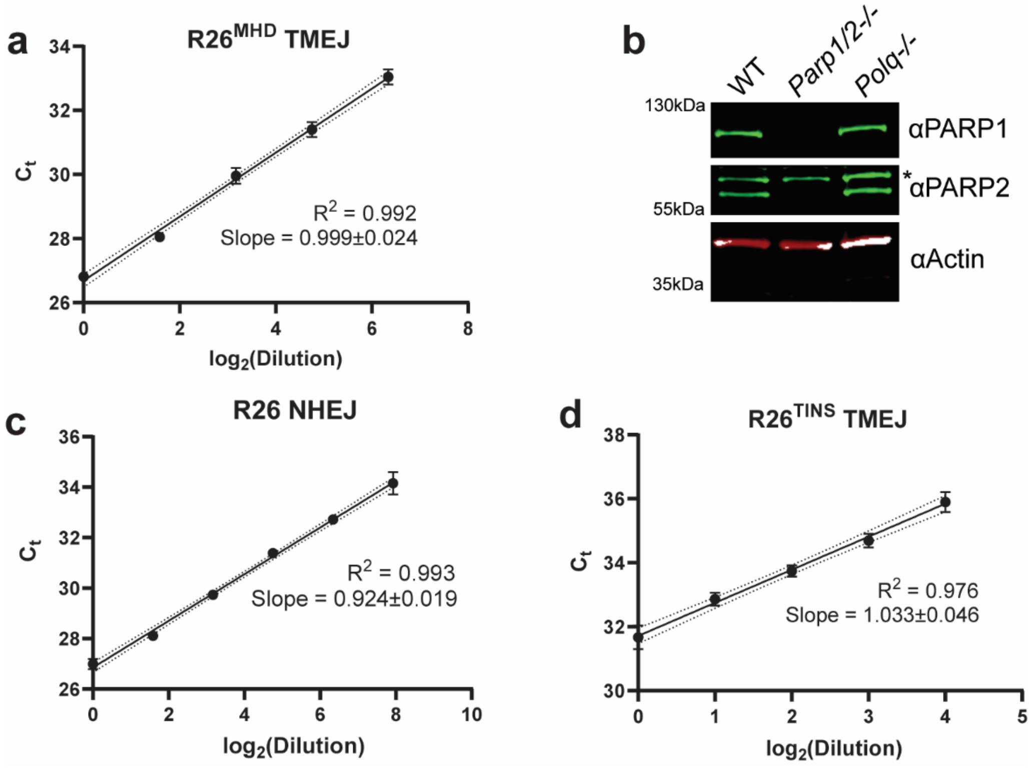

Supplementary Figure 2 Validation of linearity and limit of detection of qPCR for chromosomal repair

a) Cycle thresholds (C_t) after qPCR of R26^{MHD} TMEJ generated from WT MEFs transfected with Cas9-gRNA after serial dilution into genomic DNA from MEFs with uncut DNA. Data shown are mean ± SD (n=3); the slope, coefficient of determination (R^2), fitted line (solid line), and 95% confidence intervals (dashed lines) after simple linear regression are noted. **b)** Immunoblot for PARP1 (116 kilodaltons; kDA), PARP2 (62kDa), and actin (42kDa, loading control) in WT, *Parp1/2-/-*, and *Polq-/-* MEFs (n=2). **c)** Cycle thresholds (C_t) after qPCR for R26 NHEJ generated from WT MEFs transfected with Cas9-gRNA after serial dilution into genomic DNA from MEFs with uncut DNA. Data shown are mean ± SD (n=3); the slope, coefficient of determination (R^2), fitted line (solid line), and 95% confidence intervals (dashed lines) after simple linear regression are noted. **d)** Cycle thresholds (C_t) after qPCR of R26^{TINS} TMEJ generated from WT MEFs transfected with Cas9-gRNA after serial dilution into genomic DNA from MEFs with uncut DNA, after correction using C_ts for Ref2 (Supplementary Table 3). Data shown are mean ± SD (n=3). The slope, coefficient of determination (R^2), fitted line (solid line), and 95% confidence intervals (dashed lines) after simple linear regression are noted.

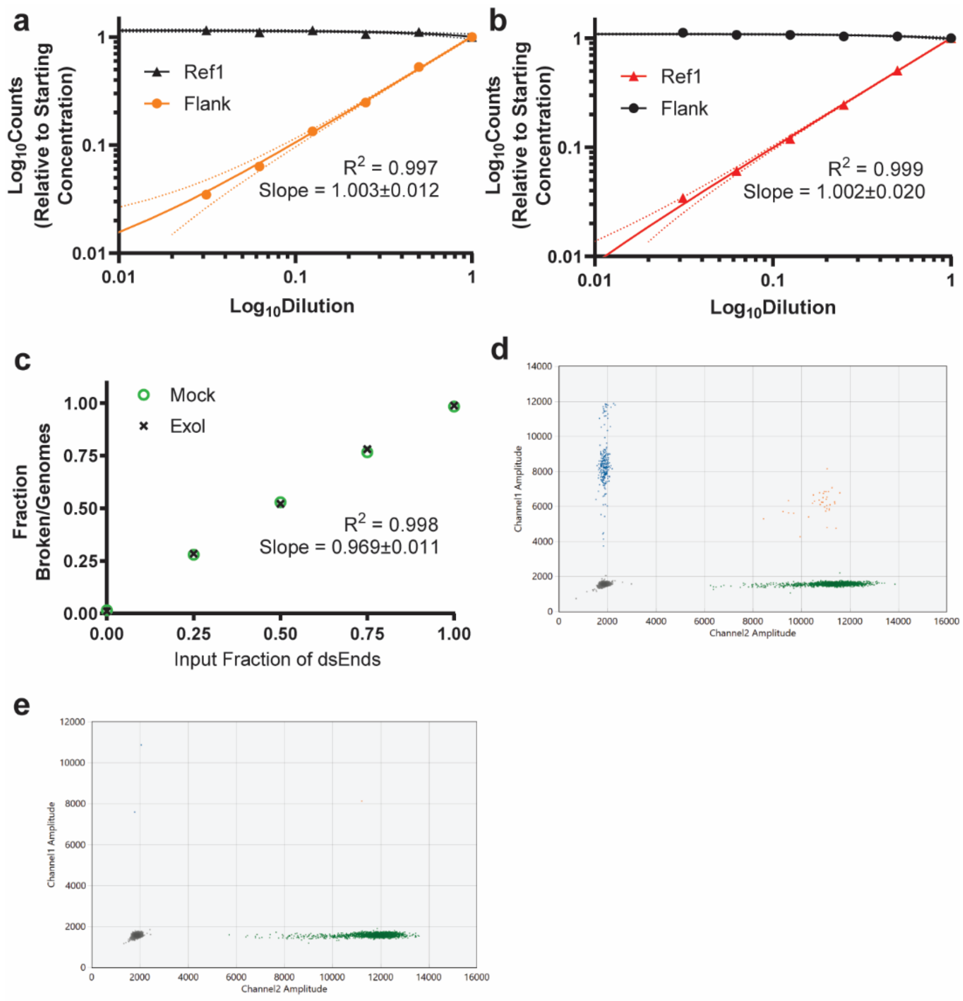

Supplementary Figure 3 Validation of linearity, limit of detection, and independence of multiplexed ddPCR

a) Flank counts, relative to the starting concentration, in samples with the flank control (ssDNA control; Supplementary Table 3) serially diluted in DNA with a constant amount of the Ref1 control. The slope, coefficient of determination (R^2), fitted line (solid orange line), and 95% confidence intervals (dashed orange lines) after simple linear regression are noted. **b)** Ref1 counts, relative to the starting concentration, in samples with the Ref1 control serially diluted in DNA with a constant amount of the flank control. The slope, coefficient of determination (R^2), fitted line (solid red line), and 95% confidence intervals (dashed red lines) after simple linear regression are noted. **c)** Fraction of broken chromosomes, relative to the number of genomes determined by Flank counts, in samples mock (green circles) or ExoI-treated (black x's) with increasing levels of chromosomes broken by digestion with XbaI (cuts adjacent to Cas9 site; input dsEnds). **d and e)** Example 2D-plot of the ssDNA control and Ref1 multiplexed-ddPCR in mock- **(d)** and ExoI-treated **(e)** samples. Blue points (channel 1/FAM) are droplets positive for only the ssDNA control, and green points (channel 2/HEX) are droplets positive for only Ref1. Droplets positive for both are orange, and double negative droplets are grey.

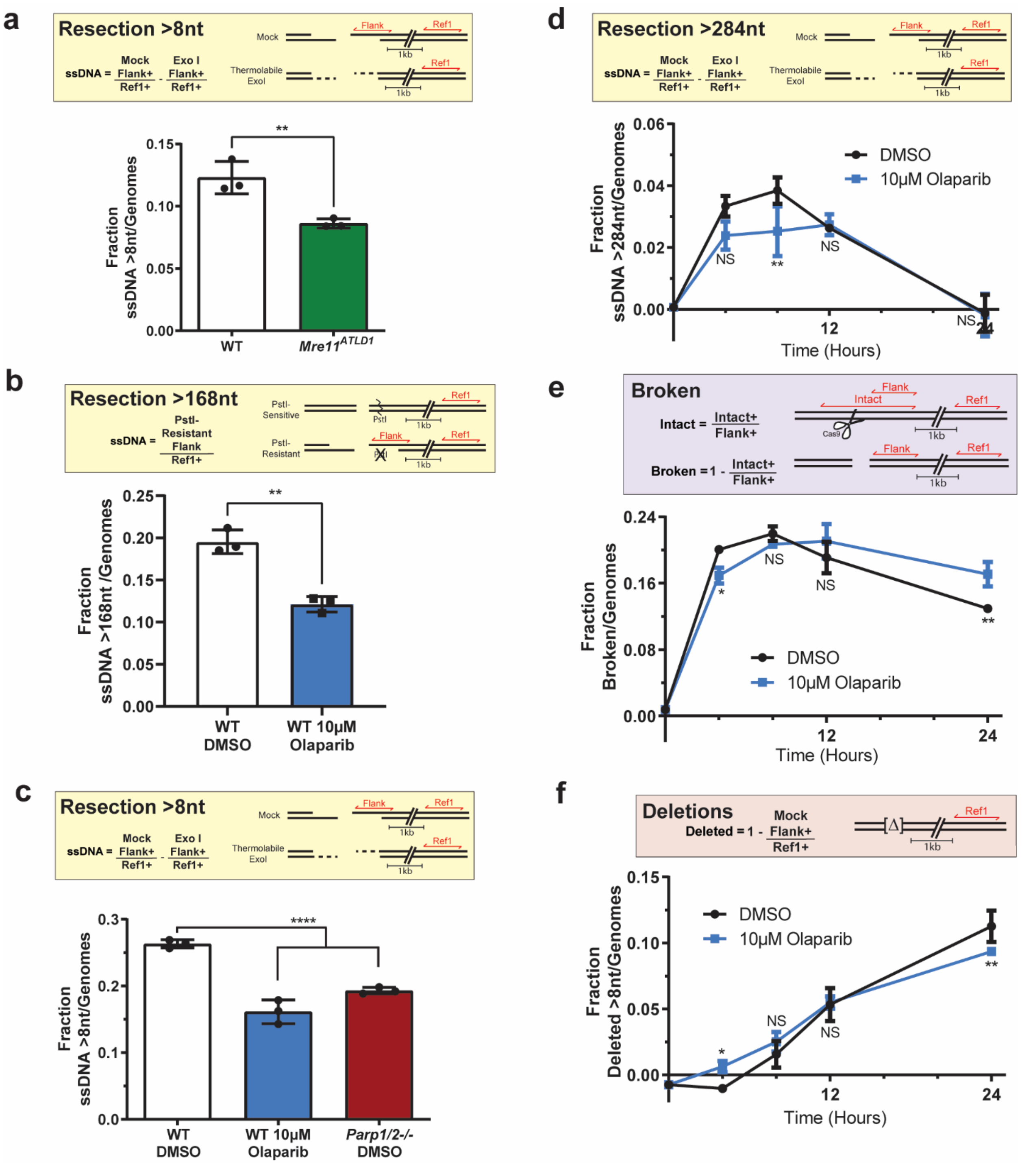

Supplementary Figure 4 PARPi does not affect broken chromosomes or deletions >8bp

a) Fraction of chromosomes with ssDNA >8nt after 4 hours in WT or *Mre*^{ATLD1} MEFs (n=3). Data shown are mean ± SD, and significance of difference determined by unpaired, two-tailed t-test (**p=0.0092). **b)** Fraction of ssDNA >168nt after 4 hours in WT MEFs treated with DMSO or 10μM olaparib as determined by PstI resistance. Data shown are mean ± SD, and significance of difference determined by unpaired, two-tailed t-test (**p=0.0016). **c)** Fraction of chromosomes with ssDNA >8nt after 4 hours in WT MEFs treated with DMSO or 10μM olaparib

or *Parp1/2-/-* MEFs (n=3). Data shown are mean ± SD, and statistical significance determined by one-way ANOVA (****p<0.0001). The same biological replicates were used in b and c. **d-f)** Fraction of ssDNA >284nt generated by long-range 5'-to-3' resection **(d)**, broken chromosomes **(e)** or deletions >8bp **(f)** in WT MEFs treated with DMSO (black) or 10µM olaparib (blue) over 24 hours. Time course data shown are mean ± SD of one biological replicate and four (d) or three (e and f) technical replicates; these data were generated from the same biological replicate used in Figure 5. Statistical significance of difference, relative to WT DMSO, was determined by one-way ANOVA (*p<0.03; **p<0.01; NS, p>0.09, not significant). Colored boxes refer to Figure 5a. Amplicons used for ddPCR are shown in red; Δ indicates deletion repair products. The number of genomes in each PCR was determined by a reference amplicon ~1kb (Ref1) from the cut site.

Supplementary Tables

Supplementary Table 1

Gene	gRNA sequence + PAM	Chr. location
Polq	ATACGGGAGCGGATGTGTTCAGG	16: 36,906,960
Polq	CAGTTCAAGCTGAGAGTAGTCGG	16: 36,906,888
Parp1	CTCAACATCAGGCTGCCGGATGG	1: 180,401,284
Parp2	CTTCAAGAGCGATGGCGCCGCGG	14: 51,045,394

gRNA sequences + PAM (underlined) and sequence location in the mouse genome for sites mutated to knock out *Polq* in KPB13 cells and knock out *Parp1* and *Parp2* in MEFs.

Supplementary Table 2

Substrate	Side	Sequence
TMEJ	Top	CGACCTTTTTGGTCGTTTTCTCACACCCATCGTACATCATTCGTCTCT<br>ATGGACCCGGCAGTGGGATCCTGACGCTGAGGTTACGGCAGTGCG<br>TGAGTTCGGTATAGTATGGTACTAAGCGATGCTCTCACCGAGCCGT<br>ATCTGCTGGGTTGTGGATGAATTACATATGCTGGGAGAACCAAGATT<br>GGGCAGTTTTT
	Bottom	CGACCTTTTTGGTCGTTTGCATCGCTTAGTACCATACTATACCGAAC<br>TCACGCACTGCCGTAACCTCAGCGTCAGGATCCCACTGCCGGGTCC<br>ATAGAGACGAATGATGTACGATGGGTGTGAGAGTGAAGATCCTCAC<br>CTTCGGAGTACTCCTTCTTTTGACCATTGATACGATACTTCTCAGCC<br>GAGCTGCTTTTT
NHEJ	Top	GACACCTTAGCTGTATAGTCACCCTGCAGAACTATCGAATAGCACGA<br>TTCACTCTGTTCCATGATCTTCACTCTCACACCCATCAGCAGTGGGA<br>CTTCGGCTGAGGAGGACACTGCTGTTAGACTTGTGGTGGATGACCT<br>AAGCGATGCTCTCACCGAGGATTATCGAGCAAGAAGCAGGGTAGCC<br>AGTCTGAGAATCGA
	Bottom	GATTCTCAGACTGGCTACCCTGCTTCTTGCTCGATAATCCTCGGTGA<br>GAGCATCGCTTAGGTCATCCACCACAAGTCTAACAGCAGTGTCCTC<br>CTCAGCCGAAGTCCCACTGCTGATGGGTGTGAGAGTGAAGATCATG<br>GAACAGAGTGAATCGTGCTATTCGATAGTTCTGCAGGGTGACTATAC<br>AGCTAAGGTGTCGA

Top and bottom strands were annealed to make extrachromosomal substrates. The microhomology is underlined in the TMEJ substrate.

Supplementary Table 3

Amplicon	Primer	Sequence
EC NHEJ	Fwd	TAAGCGATGCTCTCACCGA
	Rev	GATGGGTGTGAGAGTGAAGATC
	Probe	/5HEX/TCTCAGACT/ZEN/GGCTACCCTGCTTCT/3IABkFQ/
EC TMEJ	Fwd	TAAGCGATGCTCTCACCGA
	Rev	GATGGGTGTGAGAGTGAAGATC
	Probe	/56-FAM/CCAAGATTG/ZEN/GGCAGCTCGGC/3IABkFQ/
Ref2	Fwd	GGGAAGTGAGAGAGAAACTGAAG
	Rev	AAACCTGAGCCAGACTTTCC
	Probe	/5HEX/TCAGCAAAG/ZEN/ACCGCGGAAAGATCT/3IABkFQ/
$R26^{MHD}$ TMEJ	Fwd	TCAGTTGGGCTGTTTTGGAG
	Rev	TAAGCCTGCCCAGAAGAGTG
	Probe	/56-FAM/TCAGTAAGG/ZEN/GAGCTGCAGTGGAGTA/3IABkFQ/
R26 NHEJ	Fwd	TCAGTTGGGCTGTTTTGGAG
	Rev	AAGACTCCCGCCCATCATT
	Probe	/56-FAM/TCAGTAAGG/ZEN/GAGCTGCAGTGGAGTA/3IABkFQ/
$R26^{TINS}$ TMEJ	Fwd	CCAGCTACAGCCTCAACAC
	Rev	TACTGGCTTATCCAACCCCT
	Probe	/56-FAM/TCAGGAAAG/ZEN/GGAAAATGCCAATGCTC/3IABkFQ/
Ref1	Fwd	GTGGAGCCGTTCTGTGAG
	Rev	GCATTCCTGCCCACCAC
	Probe	/5HEX/TTCCAGCGT/ZEN/CACGACTCGTACC/3IABkFQ/
Ref1 Control	Fwd	GTGGAGCCGTTCTGTGAG
	Rev	GCATTCCTGCCCACCAC
	Probe	/5HEX/TCTTTGCCT/ZEN/TGGAGAGTGCCAGAA/3IABkFQ/
Flank 8bp	Fwd	TGCCTCCTGGCTTCTGA
	Rev	AGACTGGAGTTGCAGATCAC
	Probe	/56-FAM/AAGGGATTC/ZEN/TCCCAGGCCCA/3IABkFQ/
Flank 284bp	Fwd	CGCAACGTGGCAGGAAG
	Rev	GGAGGTCTGGCTCAGCA
	Probe	/56-FAM/ACGTTTCCG/ZEN/ACTTGAGTTGCCTCA/3IABkFQ/
Flank 527bp	Fwd	GGAGGGTCAGCGAAAGTAG
	Rev	ATGGCAAGGGCCAGTTT
	Probe	/56-FAM/AGAGCCAAT/ZEN/CAGACGACGAGGC/3IABkFQ/
Flank PstI	Fwd	TCAGTTGGGCTGTTTTGGAG
	Rev	CTGCAGCTCCCTTACTGATAAC
	Probe	/56-FAM/ACTTGCTCT/ZEN/CCCAAAGTCGCTCTG/3IABkFQ/
ssDNA Control	Oligo	TGCCTCCTGGCTTCTGACCTCAGCCTCTCAGACTGGCTACCCT GCTTCTACTTCTTCACTGAATGTGATCTGCAACTCCAGTCT
	Fwd	TGCCTCCTGGCTTCTGA
	Rev	AGACTGGAGTTGCAGATCAC
	Probe	/56-FAM/TCTCAGACT/ZEN/GGCTACCCTGCTTCT/3IABkFQ/
Intact	Fwd	TGCCTCCTGGCTTCTGA
	Rev	CAGGACAACGCCCACAC
	Probe	/56-FAM/TTTAAGCCT/ZEN/GCCCAGAAGACTCCC/3IABkFQ/
Gene Targeting	Fwd	AGAACTGCAGTGTTGAGGCC
	Rev	/5Cy5/AGAAAACTGGCCCTTGCCATT

Forward and reverse primers and probes used to quantify and characterize repair intermediates and outcomes.

Supplementary Table 4

Locus	gRNA sequence + PAM	Chr. 6 location
$R26^{MHD}$/R26 NHEJ	ACTCCAGTCTTTCTAGAAGATGG	113,068,731
$R26^{TINS}$	CCACAAATCGAGGCTGTAGCTGG	113,069,295

gRNA sequences + PAM (underlined) and sequence location in the mouse chromosome 6 for both break sites tested.

Supplementary Table 5

Target	Catalog Number	Dilution
PARP1	Enzo ALX-210-302-R100	1:2000
PARP2	Enzo ALX-210-899-R100	1:5000
Pan Actin	Novus NB600-535	1:5000
IRDye® 680LT Goat anti-Mouse IgG	LI-COR 926-68020	1:10,000
IRDye® 800CW Goat anti-Rabbit IgG	LI-COR 926-32211	1:10,000
CtIP	Novus NB-79810	1:500
gamma-H2AX	Cell Signaling Technology 9718	1:5000
Goat anti-Mouse IgG (H+L) Highly Cross -Adsorbed Secondary Antibody, Alexa Fluor 488	Invitrogen A11029	1:1000
Goat anti-Rabbit IgG (H+L) Highly Cross - Adsorbed Secondary Antibody, Alexa Fluor 488	Invitrogen A11008	1:1000

Antibodies used for immunoblotting and immunofluorescence.

Supplementary Table 6

	FAM Amplicon	FAM counts (mock)	FAM Counts (Exo I)	% Exo I Sensitive
**100% Intact**	Flank	158.5±5.5	156.5±5.5	1.2±3.5%
**50% dsEnds**	Flank	200.9±9.5	205.1±2.1	-2.1±1.0%
**Low ssEnds**	ssDNA Control	19.5±0.7	0.2±0.1	99.0±0.3%
**High ssEnds**	ssDNA Control	307.4±0.6	2.1±0.1	99.3±0.02%

Flank or ssDNA Control counts and their sensitivity to Exo I treatment in DNA with varying levels of unbroken (Intact), double-stranded break ends (dsEnd; XbaI-generated breaks) or single-stranded ends (ssEnds; using spike-in of ssDNA control).

Supplementary Table 7

PCR	Sample	Primer	SEQUENCE
R26	WT DMSO (1)	Fwd	CTACACGACGCTCTTCCGATCTACTTGAAGTTCTCT GCTGCCTCCTGGCTTCT
		Rev	GCTGAACCGCTCTTCCGATCTCGATGTCGATCTGT GGGAAGTCTTGTCCCTCCAA
	WT DMSO (2)	Fwd	CTACACGACGCTCTTCCGATCTACTTGAAGTTCTCT GCTGCCTCCTGGCTTCT
		Rev	GCTGAACCGCTCTTCCGATCTATCACGATGATCTGT GGGAAGTCTTGTCCCTCCAA
	WT DMSO (3)	Fwd	CTACACGACGCTCTTCCGATCTACTTGAAGTTCTCT GCTGCCTCCTGGCTTCT
		Rev	GCTGAACCGCTCTTCCGATCTTTAGGCTGCGATCT GTGGGAAGTCTTGTCCCTCCAA
	WT 0.1μM Olaparib (1)	Fwd	CTACACGACGCTCTTCCGATCTCAGATCTAGTTCTC TGCTGCCTCCTGGCTTCT
		Rev	GCTGAACCGCTCTTCCGATCTCGATGTCGATCTGT GGGAAGTCTTGTCCCTCCAA
	WT 0.1μM Olaparib (2)	Fwd	CTACACGACGCTCTTCCGATCTCAGATCTAGTTCTC TGCTGCCTCCTGGCTTCT
		Rev	GCTGAACCGCTCTTCCGATCTATCACGATGATCTGT GGGAAGTCTTGTCCCTCCAA
	WT 0.1μM Olaparib (3)	Fwd	CTACACGACGCTCTTCCGATCTCAGATCTAGTTCTC TGCTGCCTCCTGGCTTCT
		Rev	GCTGAACCGCTCTTCCGATCTTTAGGCTGCGATCT GTGGGAAGTCTTGTCCCTCCAA
	WT 10μM Olaparib (1)	Fwd	CTACACGACGCTCTTCCGATCTTGACCAGTAGTTCT CTGCTGCCTCCTGGCTTCT
		Rev	GCTGAACCGCTCTTCCGATCTCGATGTCGATCTGT GGGAAGTCTTGTCCCTCCAA
	WT 10μM Olaparib (2)	Fwd	CTACACGACGCTCTTCCGATCTTGACCAGTAGTTCT CTGCTGCCTCCTGGCTTCT
		Rev	GCTGAACCGCTCTTCCGATCTATCACGATGATCTGT GGGAAGTCTTGTCCCTCCAA
	WT 10μM Olaparib (3)	Fwd	CTACACGACGCTCTTCCGATCTTGACCAGTAGTTCT CTGCTGCCTCCTGGCTTCT
		Rev	GCTGAACCGCTCTTCCGATCTTTAGGCTGCGATCT GTGGGAAGTCTTGTCCCTCCAA
	Polq-/- DMSO (1)	Fwd	CTACACGACGCTCTTCCGATCTCGATGTCGAAGTT CTCTGCTGCCTCCTGGCTTCT
		Rev	GCTGAACCGCTCTTCCGATCTCGATGTCGATCTGT GGGAAGTCTTGTCCCTCCAA
	Polq-/- DMSO (2)	Fwd	CTACACGACGCTCTTCCGATCTCGATGTCGAAGTT CTCTGCTGCCTCCTGGCTTCT
		Rev	GCTGAACCGCTCTTCCGATCTATCACGATGATCTGT GGGAAGTCTTGTCCCTCCAA
	Polq-/- DMSO (3)	Fwd	CTACACGACGCTCTTCCGATCTCGATGTCGAAGTT CTCTGCTGCCTCCTGGCTTCT

		Rev	GCTGAACCGCTCTTCCGATCTTTAGGCTGCGATCT GTGGGAAGTCTTGTCCCTCCAA
	Polq-/- 10μM Olaparib (1)	Fwd	CTACACGACGCTCTTCCGATCTATCACGATGAAGTT CTCTGCTGCCTCCTGGCTTCT
		Rev	GCTGAACCGCTCTTCCGATCTCGATGTCGATCTGT GGGAAGTCTTGTCCCTCCAA
	Polq-/- 10μM Olaparib (2)	Fwd	CTACACGACGCTCTTCCGATCTATCACGATGAAGTT CTCTGCTGCCTCCTGGCTTCT
		Rev	GCTGAACCGCTCTTCCGATCTATCACGATGATCTGT GGGAAGTCTTGTCCCTCCAA
	Polq-/- 10μM Olaparib (3)	Fwd	CTACACGACGCTCTTCCGATCTATCACGATGAAGTT CTCTGCTGCCTCCTGGCTTCT
		Rev	GCTGAACCGCTCTTCCGATCTTTAGGCTGCGATCT GTGGGAAGTCTTGTCCCTCCAA
Secondary NGS PCR	All	Fwd	AATGATACGGCGACCACCGAGATCTACACTCTTTC CCTACACGACGCTCTTCCGATCT
		Rev	CAAGCAGAAGACGGCATACGAGATCGGTCTCGGC ATTCCTGCTGAACCGCTCTTCCGATCT

Forward and reverse primers used for next-generation sequencing library preparation.

Supplementary Table 8

Sample	Biological Replicate	Number of Reads in Library	Number of Reads Analyzed
WT DMSO	1	150455	149056
	2	265529	262848
	3	198029	196057
WT 0.1μM Olaparib	1	102117	101211
	2	290784	288083
	3	275601	272909
WT 10μM Olaparib	1	199878	198084
	2	261011	258575
	3	191377	189694
Polq-/- DMSO	1	121184	120511
	2	252810	251128
	3	158836	157772
Polq-/- 10μM Olaparib	1	204029	202763
	2	41477	41123
	3	123554	122808

Number of reads generated in each next-generation sequencing library and the number of reads used in the final analysis.

Supplementary Table 9

Repair Product	Deletion	WT DMSO	WT 0.1μM Olaparib	WT 10μM Olaparib	Polq-/- DMSO	Polq-/- 10μM Olaparib	Repair Pathway
TCTTTCTAGAGATGGGCGGG	1	30.9±1.5%	31.1±1.5%	25.4±1.8%	32.9±1.1%	27.9±1.5%	NHEJ
TCTTTCTAGA**A**AGATGGGCGG	0	18.0±0.7%	20.8±0.4%	26.2±0.2%	21.1±0.1%	26.6±0.8%	NHEJ
TCTTTCTAGATGGGCGGGAG	3	14.1±0.7%	12.1±0.5%	11.8±0.6%	11.4±0.3%	10.8±0.2%	TMEJ
CTCCAGTCTTCTGGGCAGGC	23	3.8±0.6%	2.7±0.5%	1.8±0.2%	0.9±0.1%	0.7±0.2%	TMEJ
AGTCTTTCTAAGATGGGCGG	2	3.8±0.1%	3.7±0.3%	3.9±0.1%	5.2±0.5%	5.2±0.1%	NHEJ
CCAGTCTTTCAGATGGGCGG	4	2.2±0.2%	2.1±0.2%	2.1±0.3%	2.5±0.1%	2.2±0.1%	NHEJ
TCTTTCTAGAATGGGCGGGA	2	1.6±0.0%	1.8±0.2%	2.2±0.2%	1.5±0.1%	2.1±0.2%	NHEJ
TCCAGTCTTTAGATGGGCGG	5	1.0±0.1%	0.8±0.1%	1.0±0.1%	1.1±0.0%	1.0±0.2%	Other
GCAACTCCAGATGGGCGGGA	12	0.9±0.1%	0.7±0.0%	0.9±0.0%	0.4±0.1%	0.4±0.0%	TMEJ
TCTTTCTAGA**T**AGATGGGCGG	0	0.8±0.1%	1.0±0.0%	1.0±0.1%	1.2±0.0%	1.4±0.1%	NHEJ
AGTCTTTCTA**A**AGATGGGCGG	2	0.8±0.1%	0.8±0.1%	0.8±0.1%	1.0±0.1%	1.1±0.2%	NHEJ
CTCCAGTCTTAGATGGGCGG	6	0.7±0.1%	0.7±0.1%	0.6±0.1%	0.7±0.1%	0.7±0.1%	Other
TCTTTCTAGA**C**AGATGGGCGG	0	0.6±0.1%	0.7±0.1%	0.8±0.0%	0.8±0.1%	0.9±0.1%	NHEJ
CCAGTCTTTC**A**AGATGGGCGG	4	0.6±0.1%	0.6±0.1%	0.6±0.0%	0.7±0.0%	0.6±0.0%	NHEJ
AGTCTTTCTATGGGCGGGAG	5	0.6±0.0%	0.5±0.1%	0.5±0.0%	0.7±0.1%	0.6±0.0%	Other
ACTCCAGTCTAGATGGGCGG	7	0.5±0.2%	0.5±0.0%	0.6±0.1%	0.6±0.0%	0.5±0.0%	Other
TCTTTCTAGA**G**AGATGGGCGG	0	0.5±0.0%	0.4±0.0%	0.5±0.0%	0.6±0.1%	0.7±0.1%	NHEJ
CAGTCTTTCTGGGCGGGAGT	7	0.5±0.1%	0.4±0.0%	0.4±0.1%	0.3±0.0%	0.3±0.1%	Other
CAGTCTTTCTGGGCAGGCTT	22	0.4±0.1%	0.4±0.1%	0.3±0.0%	0.2±0.1%	0.1±0.0%	TMEJ
TCTTTCTAGAGTCTTCTGGG	12	0.4±0.1%	0.4±0.1%	0.5±0.0%	0.3±0.0%	0.3±0.1%	Other
TCTTTCTAGAGGGCGGGAGT	4	0.4±0.1%	0.5±0.1%	0.4±0.1%	0.4±0.1%	0.4±0.1%	NHEJ
TCTTTCTAGAGCGGGAGTCT	6	0.3±0.0%	0.4±0.0%	0.5±0.0%	0.3±0.1%	0.3±0.0%	Other
TCCAGTCTTT**A**AGATGGGCGG	5	0.2±0.1%	0.3±0.1%	0.3±0.1%	0.4±0.1%	0.3±0.1%	Other
TCTTTCTAGACGGGAGTCTT	7	0.2±0.1%	0.3±0.0%	0.5±0.1%	0.2±0.0%	0.3±0.1%	Other
GTCTTTCTAGGGCGGGAGTC	6	0.2±0.0%	0.2±0.0%	0.2±0.0%	0.2±0.0%	0.2±0.0%	Other

Frequency and repair pathway designation of repair products in all cell conditions ranked by the frequency in WT DMSO cells. Repair products are identified by the sequence of the 10bp upstream followed by the 10bp downstream of the DSB. Microhomologies are underlined and insertions are bolded.
