# Supplementary material for: Poly(ADP) ribose polymerase promotes DNA polymerase theta-mediated end joining by activation of end resection
Source: Nat Commun. 2022 Aug 4;13:4547. doi: 10.1038/s41467-022-32166-7 (PMC9352658; doi:10.1038/s41467-022-32166-7)
Supplement: Supplementary file 3 — Reporting Summary [file 41467_2022_32166_MOESM3_ESM.pdf]

nature portfolio

Corresponding author(s): Dale A. Ramsden

Last updated by author(s): Jul 6, 2022

Reporting Summary

Nature Portfolio wishes to improve the reproducibility of the work that we publish. This form provides structure for consistency and transparency in reporting. For further information on Nature Portfolio policies, see our Editorial Policies and the Editorial Policy Checklist.

Statistics

For all statistical analyses, confirm that the following items are present in the figure legend, table legend, main text, or Methods section.

n/a	Confirmed	
☐	☒	The exact sample size (*n*) for each experimental group/condition, given as a discrete number and unit of measurement
☐	☒	A statement on whether measurements were taken from distinct samples or whether the same sample was measured repeatedly
☐	☒	The statistical test(s) used AND whether they are one- or two-sided<br>*Only common tests should be described solely by name; describe more complex techniques in the Methods section.*
☒	☐	A description of all covariates tested
☐	☒	A description of any assumptions or corrections, such as tests of normality and adjustment for multiple comparisons
☐	☒	A full description of the statistical parameters including central tendency (e.g. means) or other basic estimates (e.g. regression coefficient) AND variation (e.g. standard deviation) or associated estimates of uncertainty (e.g. confidence intervals)
☐	☒	For null hypothesis testing, the test statistic (e.g. *F*, *t*, *r*) with confidence intervals, effect sizes, degrees of freedom and *P* value noted<br>*Give P values as exact values whenever suitable.*
☒	☐	For Bayesian analysis, information on the choice of priors and Markov chain Monte Carlo settings
☒	☐	For hierarchical and complex designs, identification of the appropriate level for tests and full reporting of outcomes
☒	☐	Estimates of effect sizes (e.g. Cohen's *d*, Pearson's *r*), indicating how they were calculated

Our web collection on statistics for biologists contains articles on many of the points above.

Software and code

Policy information about availability of computer code

Data collection	QuantStudios 1.7.1; QX Manager 1.2 and 2.0; Volicity 6.3, TyphoonFLA9500
Data analysis	Microsoft Excel (Version 2112); GraphPad Prism 9.3.1; QuantStudios 1.7.1 ; QX Manager 1.2 Standard Edition; CLC Genomics Workbench 12.0; Imaris 9.5; FIJI 2.1.0; ImageQuant TL Toolbox 8.2<br>Custom code described in methods and software reporting policy and is uploaded at https://github.com/aluthman/Ramsden-Lab.git

For manuscripts utilizing custom algorithms or software that are central to the research but not yet described in published literature, software must be made available to editors and reviewers. We strongly encourage code deposition in a community repository (e.g. GitHub). See the Nature Portfolio guidelines for submitting code & software for further information.

Data

Policy information about availability of data

All manuscripts must include a data availability statement. This statement should provide the following information, where applicable:

- Accession codes, unique identifiers, or web links for publicly available datasets
- A description of any restrictions on data availability
- For clinical datasets or third party data, please ensure that the statement adheres to our policy

All raw fastq files are available at NCBI SRA accession code PRJNA806204 (https://www.ncbi.nlm.nih.gov/bioproject/PRJNA806204/). All other data generated in this study are provided in the Supplementary Information/Source Data file.

Field-specific reporting

Please select the one below that is the best fit for your research. If you are not sure, read the appropriate sections before making your selection.

☒ Life sciences ☐ Behavioural & social sciences ☐ Ecological, evolutionary & environmental sciences

For a reference copy of the document with all sections, see nature.com/documents/nr-reporting-summary-flat.pdf

Life sciences study design

All studies must disclose on these points even when the disclosure is negative.

Sample size	Sample sizes (at least 3 experimental replicates in most experiments, unless indicated otherwise) were sufficient for reporting statistical significance.
Data exclusions	We excluded sequences with junctions containing base ambiguities (i.e. N, W, S, R, K) and junctions with base substitutions in the 3-10 nucleotides proximal to the break site if nucleotides adjacent to the substitution matched the corresponding reference sequence; these substitutions are consistent with polymerase error during sample amplification and are misattributed as insertions.<br>A data point was determined to be an outlier via Grubb's test and excluded from Fig. 5b.<br>A data point was eliminated from Fig. 5d because it was less than all values at the previous time point for an event that does not decrease over time.
Replication	Details of replicates are included in the methods and figure legends.
Randomization	Our study is not subject to randomization since it does not involve allocation into experimental groups; therefore, randomization is not relevant for the experiments performed.
Blinding	Quantifiable data was obtained directly from instruments, and all samples were analyzed/transformed via identical methods; therefore, blinding was not necessary.

Reporting for specific materials, systems and methods

We require information from authors about some types of materials, experimental systems and methods used in many studies. Here, indicate whether each material, system or method listed is relevant to your study. If you are not sure if a list item applies to your research, read the appropriate section before selecting a response.

Materials & experimental systems

n/a	Involved in the study	
☐	☒	Antibodies
☐	☒	Eukaryotic cell lines
☒	☐	Palaeontology and archaeology
☒	☐	Animals and other organisms
☒	☐	Human research participants
☒	☐	Clinical data
☒	☐	Dual use research of concern

Methods

n/a	Involved in the study	
☒	☐	ChIP-seq
☒	☐	Flow cytometry
☒	☐	MRI-based neuroimaging

Antibodies

Antibodies used	Primary antibodies used for immunoblots:<br>PARP1: Enzo ALX-210-302-R100<br>PARP2: Enzo ALX-210-899-R100<br>pan Actin: Novus NB600-535<br><br>Secondary antibodies used for immunoblots:<br>IRDye® 680LT Goat anti-Mouse IgG: LI-COR 926-68020<br>IRDye® 800CW Goat anti-Rabbit IgG: LI-COR 926-32211<br><br>Primary Antibodies used for immunofluorescence:<br>CtIP: Novus NB-79810<br>gamma-H2AX: Cell Signaling Technology 9718<br><br>Secondary antibodies used for immunofluorescence:<br>Goat anti-Mouse IgG (H+L) Highly Cross -Adsorbed Secondary Antibody, Alexa Fluor 488: A11029<br>Goat anti-Rabbit IgG (H+L) Highly Cross -Adsorbed Secondary Antibody, Alexa Fluor 488: A11008

Validation	Primary antibodies used for immunoblots:<br>PARP1: Enzo ALX-210-302-R100; validation stated on supplier website https://www.enzolifesciences.com/fileadmin/reports/Datasheet-ALX-210-302.pdf<br>PARP2: Enzo ALX-210-899-R100; validation stated on supplier website https://www.enzolifesciences.com/fileadmin/reports/Datasheet-ALX-210-899.pdf<br>pan Actin: Novus NB600-535; validation stated on supplier website https://www.novusbio.com/PDFs/NB600-535.pdf<br><br>Secondary antibodies used for immunoblots:<br>IRDye® 680LT Goat anti-Mouse IgG: LI-COR 926-68020; validation stated on supplier website https://www.licor.com/bio/reagents/irdye-680lt-goat-anti-mouse-igg-secondary-antibody<br>RDye® 800CW Goat anti-Rabbit IgG: LI-COR 926-32211; validation stated on supplier website https://www.licor.com/bio/reagents/irdye-800cw-goat-anti-rabbit-igg-secondary-antibody<br><br>Primary Antibodies used for immunofluorescence:<br>CtIP: Novus NB-79810; validation stated on supplier website https://www.novusbio.com/products/ctip-antibody_nb100-79810<br>gamma-H2AX: Cell Signaling Technology 9718; validation stated on supplier website https://www.cellsignal.com/products/primary-antibodies/phospho-histone-h2a-x-ser139-20e3-rabbit-mab/9718<br><br>Secondary antibodies used for immunofluorescence:<br>Goat anti-Mouse IgG (H+L) Highly Cross -Adsorbed Secondary Antibody, Alexa Fluor 488: A11029; validation stated on supplier website https://www.thermofisher.com/antibody/product/Goat-anti-Mouse-IgG-H-L-Highly-Cross-Adsorbed-Secondary-Antibody-Polyclonal/A-11029<br>Goat anti-Rabbit IgG (H+L) Highly Cross -Adsorbed Secondary Antibody, Alexa Fluor 488: A11008; validation stated on supplier website https://www.thermofisher.com/antibody/product/Goat-anti-Rabbit-IgG-H-L-Cross-Adsorbed-Secondary-Antibody-Polyclonal/A-11008

Eukaryotic cell lines

Policy information about cell lines

Cell line source(s)	Mouse Embryonic Fibroblasts (MEFs) derived from C57BL/6 mice - a gift from Richard Wood<br>KPB13 murine mammary tumor cells - a gift from Charles Perou<br>Retinal pigment epithelial (RPE-1) - purchased from ATCC
Authentication	MEF lines were confirmed by allele sequencing and functionally for Polq (extrachromosomal assay) or by immunoblot for Parp1/2 and Mre11.<br><br>KPB13 lines were confirmed by RT-qPCR for expression of BRCA1 or functionally for Polq (extrachromosomal assay).<br><br>Halo-POLQ RPE-1 cells were confirmed by immunofluorescence, and POLQ-/- RPE were confirmed functionally (extrachromosomal assay).
Mycoplasma contamination	All cell lines were confirmed to be free of mycoplasma contamination by PCR (detection limit less than 10 genomes/mL).
Commonly misidentified lines<br>(See ICLAC register)	No commonly misidentified lines were used in this study.
